# EDTA-Assisted Synthesis of Nitrogen-Doped Carbon Nanospheres with Uniform Sizes for Photonic and Electrocatalytic Applications

**DOI:** 10.1021/acs.chemmater.3c00341

**Published:** 2023-03-28

**Authors:** Jacob Jeskey, Yidan Chen, Sujin Kim, Younan Xia

**Affiliations:** † School of Chemistry and Biochemistry, Georgia Institute of Technology, Atlanta, Georgia 30332, United States; ‡ School of Materials Science and Engineering, Georgia Institute of Technology, Atlanta, Georgia 30332, United States; § The Wallace H. Coulter Department of Biomedical Engineering, Georgia Institute of Technology and Emory University, Atlanta, Georgia 30332, United States

## Abstract

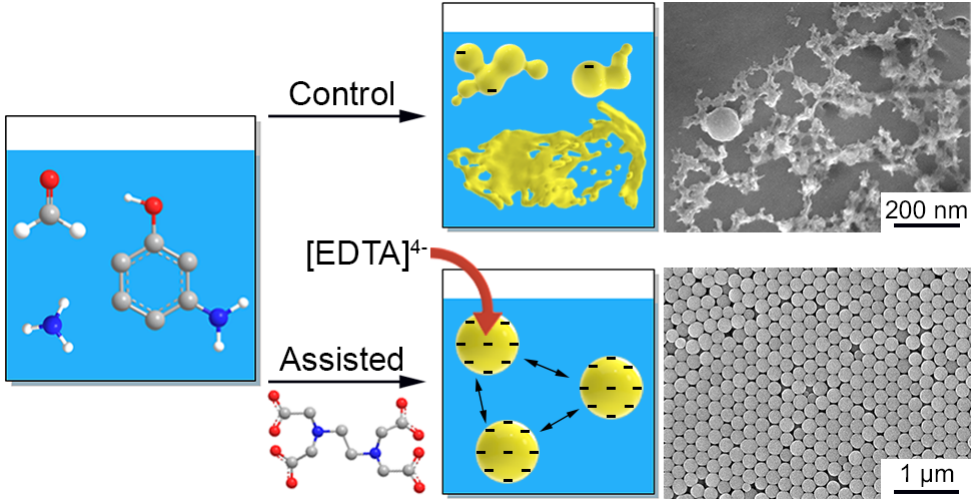

We report a robust method for the facile synthesis of N-doped carbon nanospheres with uniform and tunable sizes. Instead of involving a surfactant or other templates, this synthesis relies on the incorporation of ethylenediaminetetraacetic acid (EDTA) into the emulsion droplets of phenolic resin oligomers. The EDTA provides a high density of surface charges to effectively increase the electrostatic repulsion between the droplets and thereby prevent them from coalescing into irregular structures during polymerization-induced hardening. The EDTA-loaded polymer nanospheres are highly uniform in terms of both size and shape for easy crystallization into opaline structures. While maintaining good uniformity, the diameters of the resultant N-doped carbon nanospheres can be readily tuned from 100 to 375 nm, allowing for the fabrication of opaline lattices with brilliant structural colors. The EDTA also serves as an additional nitrogen source to promote the formation of graphitic-N, making the N-doped carbon nanospheres highly active, metal-free bifunctional electrocatalysts toward oxygen reduction and oxygen evolution reactions.

## Introduction

Uniform carbon nanospheres have gained considerable interest owing to their broad range of applications in photonics, electrocatalysis, drug delivery, and colloidal synthesis.^1−8^ In general, these applications are strongly dependent on the uniformity, size range, and surface functionality of the carbon nanospheres. For example, when carbon nanospheres are highly uniform (i.e., with size variation <5%), they can crystallize into three-dimensional opaline lattices.^9−11^ The periodicity found in such a highly ordered structure not only gives rise to interesting optical properties but also ensures sufficient mass diffusion during electrocatalysis.^12,13^ In most applications, the size of the carbon nanospheres is also equally important. Besides the advantage in specific surface area, uniform carbon nanospheres with diameters below 200 nm are highly desirable because they can be internalized by cells through endocytosis while displaying noniridescent structural colors when crystallized into opaline lattices.^4,12,14,15^ For these reasons, enormous attention has been devoted to the development of methods capable of producing uniform carbon nanospheres.

There are extensive reports on the synthesis of carbon micro-/nanospheres, but very few of them can provide samples uniform enough to crystallize into opaline lattices with tunable structural colors. In one study, Li and co-workers demonstrated an emulsion-based synthesis, where phenol and formaldehyde were polymerized using Bis-Tris as the catalyst and a surfactant such as cetyltrimethylammonium bromide (CTAB) as the emulsion stabilizer and soft template.^12^ In another report, Lu and co-workers achieved similar results with the use of resorcinol, a disubstituted phenol derivative.^10^ In both cases, the carbon nanospheres were not adequately functionalized with nitrogen (N) heteroatoms, the crucial active sites needed to achieve superior electrocatalysis and enhanced wettability.^16−19^ As a result, these carbon nanospheres would have limited use as metal-free electrocatalysts. To our knowledge, there is no report on the use of a nitrogen-containing phenolic derivative such as 3-aminophenol to produce uniform, N-doped carbon nanospheres with tunable diameters below 200 nm. This is likely due to the increased electron density that 3-aminophenol has over its derivatives, making polymerization much faster and thus limiting the ability to tailor particle size with a kinetically controlled procedure.^20^ Coincidentally, the amine group in 3-aminophenol can self-catalyze the polymerization reaction with formaldehyde, further increasing the difficulty of tailoring the synthesis to obtain particles with desirable sizes while maintaining good uniformity.^21^ Although surfactants can be added to control the particle size, they almost always resulted in morphological distortion when 3-aminophenol was used.^22−24^ Taken together, from both scientific and technological points of view, there is a pressing need to develop synthetic methods capable of producing uniform N-doped carbon nanospheres with tunable sizes below 200 nm to satisfy an array of demands from photonics, electrocatalysis, and nanomedicine.

Herein, we report a robust method for the facile synthesis of N-doped carbon nanospheres without involving a surfactant or other types of templates. The synthesis relies on the use of ethylenediaminetetraacetic acid (EDTA) as a surface stabilizing agent to prevent the emulsion nanodroplets from aggregation via electrostatic repulsion and thus improve size uniformity. The diameters of the resultant N-doped carbon nanospheres can be readily tuned from 100–375 nm without compromising uniformity, allowing them to crystallize into three-dimensional lattices with opaline colors. Moreover, owing to the high nitrogen contents and reduced particle sizes, the as-obtained carbon nanospheres display remarkable performance as metal-free electrocatalysts toward both the oxygen reduction reaction (ORR) and oxygen evolution reaction (OER).

## Experimental Section

### Chemicals and Materials

3-Aminophenol was purchased from Alfa Aesar. Formaldehyde (37 wt %), ammonium hydroxide (28–30 wt %), and EDTA were ordered from Fisher Scientific. Nafion (5 wt %) was acquired from Sigma-Aldrich. Ethanol (200 proof) was obtained from Pharmco Products. Potassium hydroxide was purchased from VWR. All aqueous solutions were prepared using deionized (DI) water with a resistivity of 18.2 MΩ·cm at room temperature.

### EDTA-Assisted Synthesis of Phenolic Resin Nanospheres

In the standard synthesis, 60 mg of 3-aminophenol was dissolved in an ethanol solution containing 40 mL of water and 16 mL of ethanol under magnetic stirring. Then, 50 mg of EDTA was introduced, followed by the addition of 0.15 mL of ammonium hydroxide to raise the pH to 9.26. Finally, 0.036 mL of 37 wt % formaldehyde was added, and the mixture was stirred for 4 h at room temperature. The as-obtained mixture was transferred into a 125 mL Teflon container and subjected to thermal treatment at 80 °C for 20 h. The resulting phenolic resin nanospheres were collected by centrifugation at 11,000 rpm for 20 min and washed with water. As a control, phenolic resin nanospheres were synthesized using the same protocol except that no EDTA was added.

### Preparation of N-Doped Carbon Nanospheres

The phenolic resin nanospheres prepared in the presence or absence of EDTA were placed in a tube furnace for thermal treatment under flowing N_2_ at 800 °C for 2 h at a heating rate of 1 °C min^–1^. The products were termed carbon nanospheres with and without EDTA assistance, or CNS-E-*X* and CNS-*X*, respectively, where “*X*” corresponds to the amount (in mg) of 3-aminophenol used in the synthesis (Table S1).

### Tailoring the Size of Polymer and N-Doped Carbon Nanospheres

The size of the phenolic resin nanospheres can be tuned in the standard protocol by varying the amounts of 3-aminophenol and formaldehyde used while their molar ratio was fixed at 1:1.2. For example, by changing the amount of 3-aminophenol to 75, 100, and 600 mg, polymer nanospheres could be produced with uniform diameters of 256, 284, and 464 nm, respectively. The diameters of the corresponding N-doped carbon nanospheres after carbonization were 178, 204, 235, and 375 nm.

### Characterizations

Scanning electron microscopy (SEM) images were obtained using a Hitachi SU-8230. Prior to SEM analysis, the samples were dispersed in ethanol at concentrations of 5–10 wt %, followed by sonication. The samples were then deposited on silicon wafers and dried at 80 °C for 1 h. Transmission electron microscopy (TEM) images were obtained on a Hitachi HT7700. Prior to TEM analysis, the samples were dispersed in ethanol at concentrations of 5–10 wt % by moderate sonication, followed by deposition on carbon-coated, 200-mesh, copper TEM grids by dipping into the sample suspension and drying at 80 °C for 1 h. A Malvern Zetasizer Nano ZS was used to measure the zeta (ζ) potentials after 4 h into the reaction. X-ray photoelectron spectroscopy (XPS) data were collected on a Thermo K-Alpha spectrometer with an Al Kα source. High-angle annular dark-field scanning TEM (HAADF-STEM) and energy dispersive X-ray (EDX) mapping images were acquired using an aberration-corrected Hitachi HD-2700 STEM. The Raman spectra were collected using a Renishaw inVia Raman spectrometer integrated with a Leica microscope. Nitrogen sorption was performed using an autoadsorption analyzer (micromeritics, 3Flex) at −196 °C. Thermogravimetric analysis (TGA) and differential scanning calorimetry (DSC) were conducted on a TGA Q600 analyzer under air at a heating rate of 10 °C min^–1^. Elemental analysis of carbon, hydrogen, nitrogen, and oxygen was conducted using a LECO TruSpec Micro elemental analyzer.

### Electrocatalytic Measurements

The electrochemical measurements were conducted at room temperature using a three-electrode cell and a CHI 600E potentiostat electrochemical workstation. A glassy carbon rotating disk electrode (RDE, 5 mm in diameter) loaded with the catalyst served as the working electrode, together with a Pt wire mesh as the counter electrode and a saturated calomel electrode (SCE) as the reference electrode. The working electrode was prepared by polishing with 0.3 μm Al_2_O_3_ powders and washing with water and ethanol. The working electrode was then polished in the same fashion using 0.05 μm Al_2_O_3_ powders. The catalyst ink was prepared by ultrasonicating 10 mg of the carbon nanospheres with a mixture containing 312.5 μL of H_2_O, 937.5 μL of ethanol, and 5 μL of 5 wt % Nafion solution for 1 h to form a homogeneous suspension. Afterward, 15 μL of the as-prepared catalyst ink was dropped on the polished RDE and dried at room temperature. As a benchmark, Pt/C catalyst ink (20 wt % Pt nanoparticles on Vulcan XC-72 carbon support, Premetek Co.) was prepared using the same protocol except 1.25 mg of the commercial catalyst was used. Before electrochemical measurements, all solutions were purged and saturated with Ar or O_2_. To measure ORR or OER activity, the catalyst was first cycled 30 times between 0.05 and 1.1 V (vs reversible hydrogen electrode, RHE) at 100 mV s^–1^ in Ar-saturated 0.1 M KOH. A background CV was obtained under the same conditions except the scan rate was reduced to 10 mV s^–1^. ORR measurements were conducted by cycling the potential 20 times between 0.1 and 1.1 V_RHE_ at 10 mV s^–1^ in O_2_-saturated 0.1 M KOH. The ORR plots were corrected for double-layer capacitance by subtracting the background CV scan. OER measurements were conducted by cycling the potential 20 times between 1.1 and 1.7 V_RHE_ at 10 mV s^–1^ in O_2_-saturated 0.1 M KOH. The OER plots were corrected for double-layer capacitance by averaging the positive and negative sweeping scans. The onset potential (*E*_onset_) was defined as the potential at which the first derivative starts increasing.

## Results and Discussion

**Figure 1:**
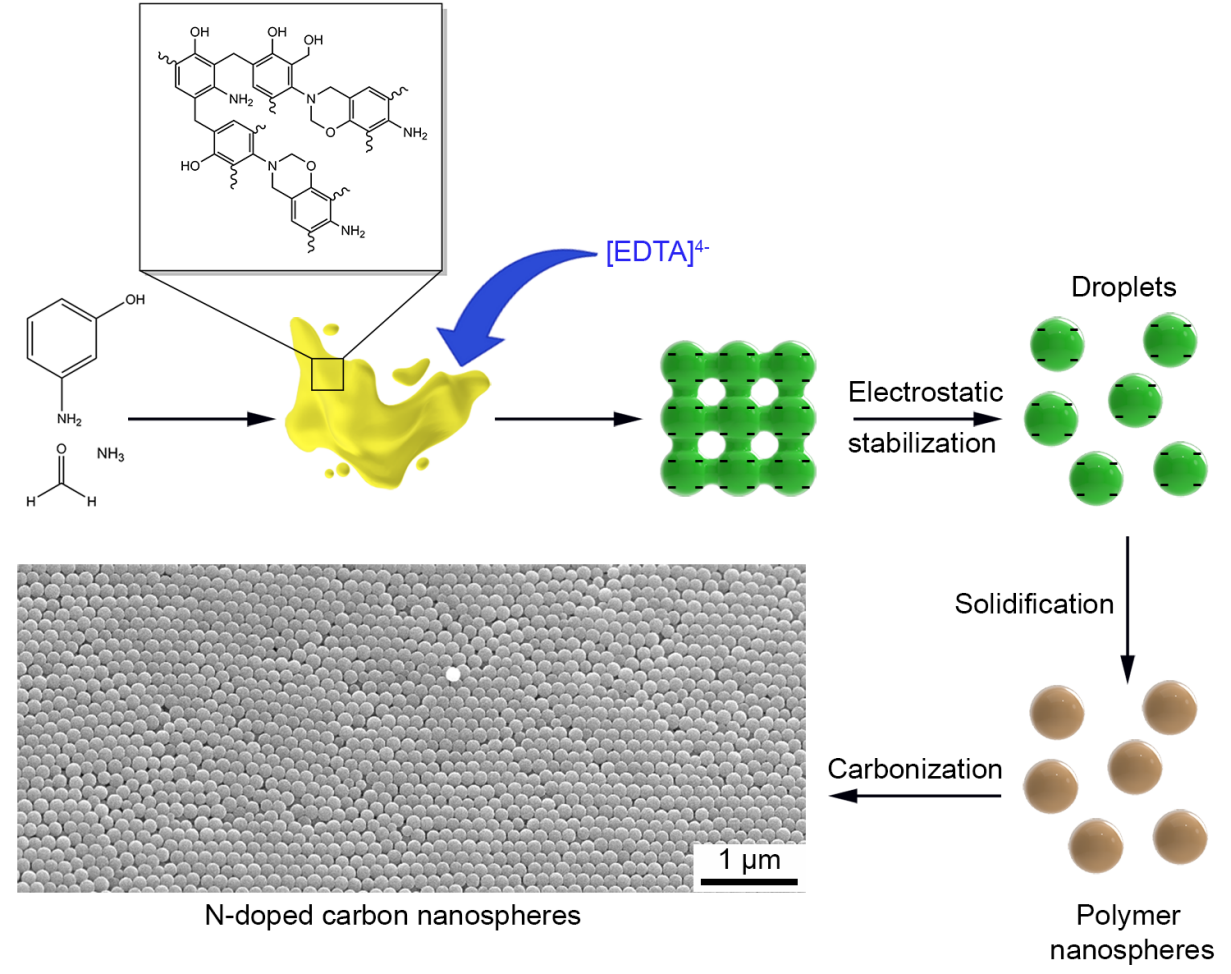
Schematic illustration showing the synthesis of N-doped carbon nanospheres through an EDTA-assisted emulsion polymerization process, followed by carbonization.

During the initial polymerization stage, 3-aminophenol and formaldehyde rapidly undergo step-growth condensation to form a variety of hydroxymethyl and benzoxazine derivatives.^11,20,21^ These intermediates then preferentially form emulsion droplets to minimize the interfacial energy between the hydrophobic oligomers and the hydrophilic medium.^25^ The formation of such droplets is very fast (typically, within 2–5 min) and consumes most of the monomers in the growth medium. As time elapses, the small oligomers that make up the droplets will be cross-linked to form a large network polymer, gradually hardening the droplets into solid polymer nanospheres. However, before cross-linking occurs, the oligomer droplets can easily coalesce into irregular structures in the absence of an emulsion stabilizer. In fact, simply stirring the emulsion at a certain speed may force the droplets into contact, resulting in severe aggregation.^26,27^ Taken together, the emulsion droplets must remain isolated from each other until they have been sufficiently hardened to certain rigidity in order to obtain polymer nanospheres with a uniform size. Here we demonstrate that it is feasible to increase the density of charges on the emulsion droplets using a highly charged species such as EDTA and thereby amplify electrostatic repulsion among the droplets (Figure 1). Because the deprotonated carboxylic acids in EDTA are negatively charged, the emulsion droplets will inevitably become increasingly charged to resist coalescence as more EDTA was uptaken.

**Figure 2:**
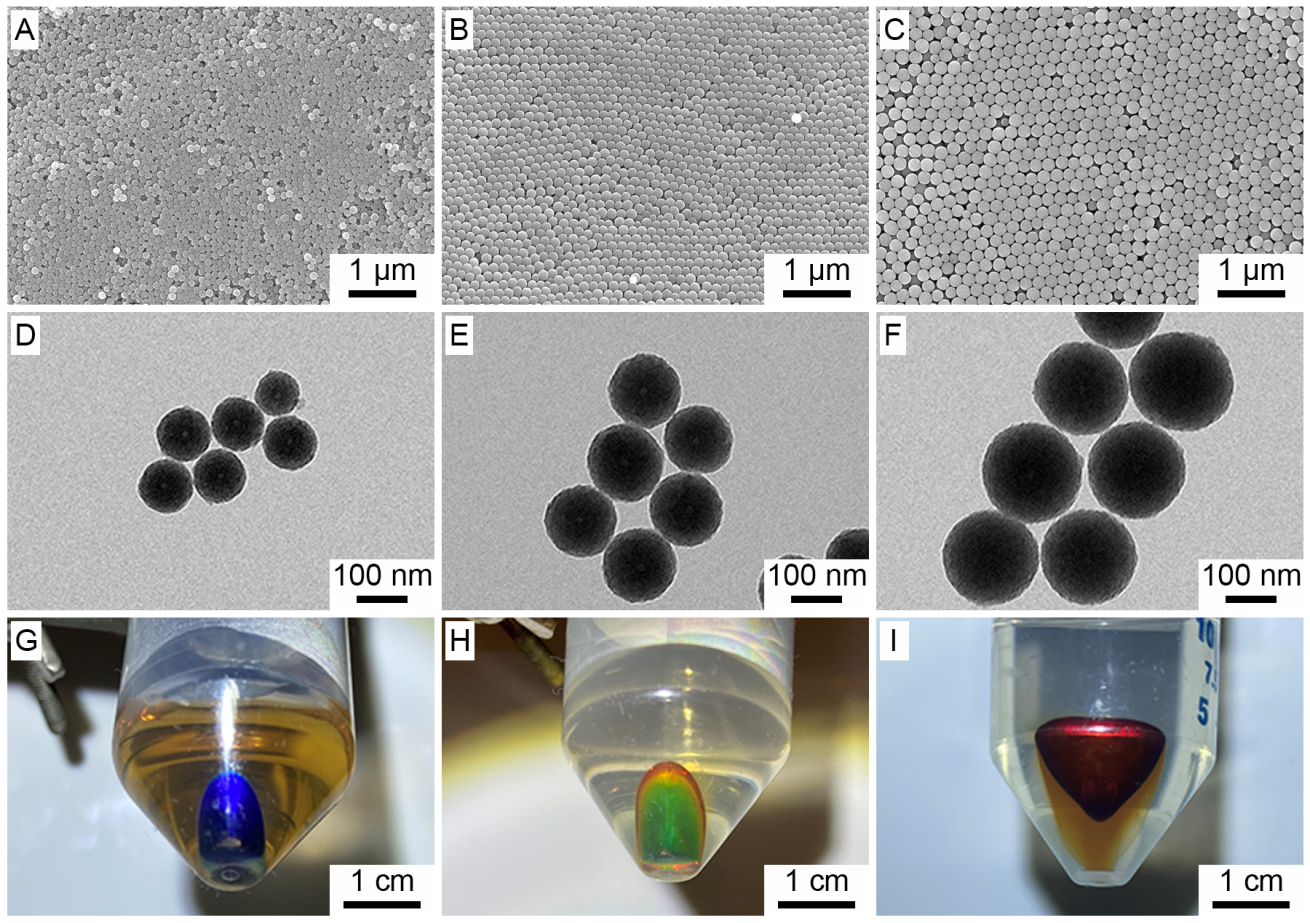
(top) SEM and (middle) TEM images of EDTA-assisted N-doped carbon nanospheres prepared using the standard procedure, except the amount of EDTA was changed to (A, D) 10 mg, (B, E) 30 mg, and (C, F) 50 mg. (bottom) Photographs of opaline structures derived from the polymer nanospheres prepared using (G) 10 mg, (H) 30 mg, and (I) 50 mg of EDTA.

Indeed, as demonstrated by the SEM and TEM images in Figure 2, the resultant N-doped carbon nanospheres were highly uniform when EDTA was incorporated into the synthesis. Interestingly, the particle size was also varied by controlling the amount of EDTA added. When the amount of EDTA was set to 10, 30, and 50 mg, the resultant N-doped carbon nanospheres were 103 ± 8, 143 ± 7, and 178 ± 8 nm, respectively, in diameter (Figure 2A–C). Even though EDTA speeded up the formation of emulsion droplets, as indicated by the rapid color change from clear to opaque during initial polymerization (Figure S1), the size of the N-doped carbon nanospheres actually increased with EDTA. This is seemingly contradictive of a faster nucleation rate, as it is well-known that acceleration in kinetics would lead to more nucleation and thus smaller particles. However, considering that EDTA is a relatively large organic molecule, it makes sense that the particle size increases with EDTA, as more space within the polymer matrix would be occupied by EDTA. Once trapped in the polymer matrix, EDTA will be carbonized along with the polymer nanospheres during thermal treatment.

The SEM and TEM images in Figure 2 confirm that the samples prepared in the presence of EDTA are highly uniform in size. Indeed, before carbonization, the polymer nanospheres could be assembled into opaline lattices with interesting optical properties. Upon centrifugation, the as-obtained opaline lattices exhibited shiny blue, green, and red colors as a result of optical diffraction from the periodic structure (Figure 2G–I). Expectedly, the color displayed by the sample varied with particle size as defined by Bragg’s law: the wavelength of light was red-shifted from blue to green to red as the diameters of the polymer nanospheres increased from 128 ± 7 to 157 ± 5 and 228 ± 18 nm, respectively.^28^ Upon carbonization at 800 °C, the N-doped carbon nanospheres experienced a 20% shrinkage in size relative to their polymer counterparts, and the samples displayed a glossy finish. For comparison, a control sample was also prepared using the same standard procedure, except no EDTA was added (Figure S2A,C). In this case, the carbon nanospheres were highly distorted and often welded together. The diameters of the carbon nanospheres synthesized without EDTA varied from 85 to 122 nm, and the majority of the sample existed as a carbon film (Figure S2A). We measured ζ-potentials to provide more insight into the forces governing the emulsion polymerization process. The polymer nanospheres prepared with EDTA using the standard protocol were highly charged, as revealed by a large ζ-potential of −51 ± 1 mV. In contrast, the control sample was less charged with a ζ-potential of −42 ± 1 mV. The superior surface charge is likely a consequence of the carboxylates grafted from EDTA onto polymer sphere surface. These results indicate that without EDTA, the emulsion droplets would lack the electrostatic repulsion needed to prevent particle cohesion, resulting in irregular structures. A similar observation was reported by Xu and co-workers, where the emulsion droplets would coalesce into poorly defined structures when they lacked surface charges.^11^

**Figure 3:**
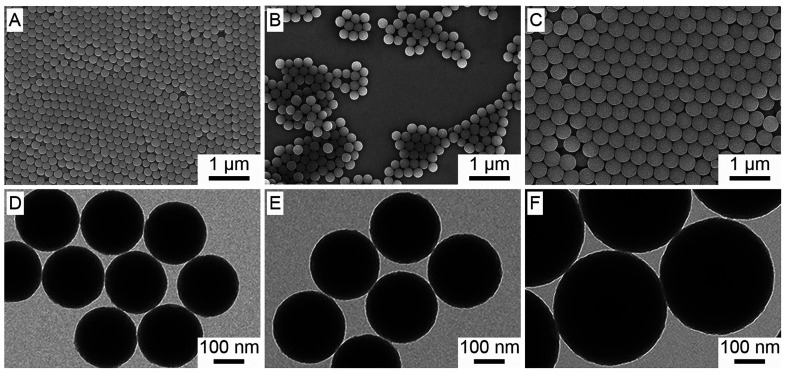
(top) SEM and (bottom) TEM images of the N-doped carbon nanospheres prepared in the presence of EDTA and at various monomer concentrations: (A, D) 75 mg, (B, E) 100 mg, and (C, F) 600 mg.

**Figure 4:**
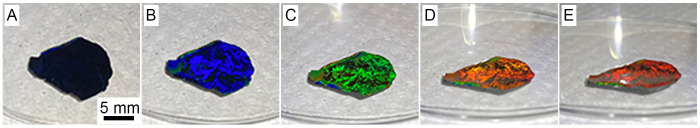
Photographs of (top) polymer and (bottom) carbon opaline lattices prepared using the standard procedure, except 600 mg of 3-aminophenol was used. Images were acquired at viewing angles of (A) 0°, (B) 30°, (C) 40°, (D) 45°, and (E) 50° (from the normal to the surface) for the polymer sample and of (F) 0°, (G) 40°, (H) 50°, (I) 60°, and (J) 65° for the carbonized sample. Scale bars are the same and displayed in (A) and (F).

The size of the N-doped carbon nanospheres could also be tuned by increasing the amount of 3-aminophenol and formaldehyde monomers. For simplicity, all other chemicals, including EDTA, were maintained at the amount specified in the standard protocol, except for the monomers which were increased at the same molar ratio. As shown by the TEM and SEM images in Figure 3, when the amount of 3-aminophenol was increased to 75, 100, and 600 mg, the average diameters of the N-doped carbon nanospheres increased to 204 ± 8, 235 ± 6, and 375 ± 4 nm, respectively. Even though the particle size was expected to increase, it was remarkable how effective EDTA was at stabilizing the emulsion, particularly in the case of CNS-E-600, where the monomer concentration was 10 times higher than what was used in the standard protocol. Regardless of the monomer concentration, all of the samples exhibited good uniformity and were capable of forming opaline lattices without involving any purification step, which is often difficult to achieve in the absence of a surfactant or other templates. CNS-E-600 was particularly interesting as the particle size was large enough to give rise play-of-color (i.e., iridescence) across the entire visible region (Figure 4).^28^ Specifically, the polymer and N-doped carbon nanospheres synthesized using 600 mg of 3-aminophenol did not show any structural color at normal incidence, and thus their true colors (brown for polymer nanospheres and black for N-doped carbon nanospheres) were observed in Figures 4A and 4F, respectively. However, by simply increasing the incident and viewing angles, the structural color could be gradually tuned across the visible region. For additional comparison, a control sample without EDTA was also prepared using 600 mg of 3-aminophenol (Figure S2B,D). As expected, the particles in CNS-600 aggregated to give a broad size distribution of 254–345 nm, and no shiny color was observed at any viewing angle.

**Figure 5:**
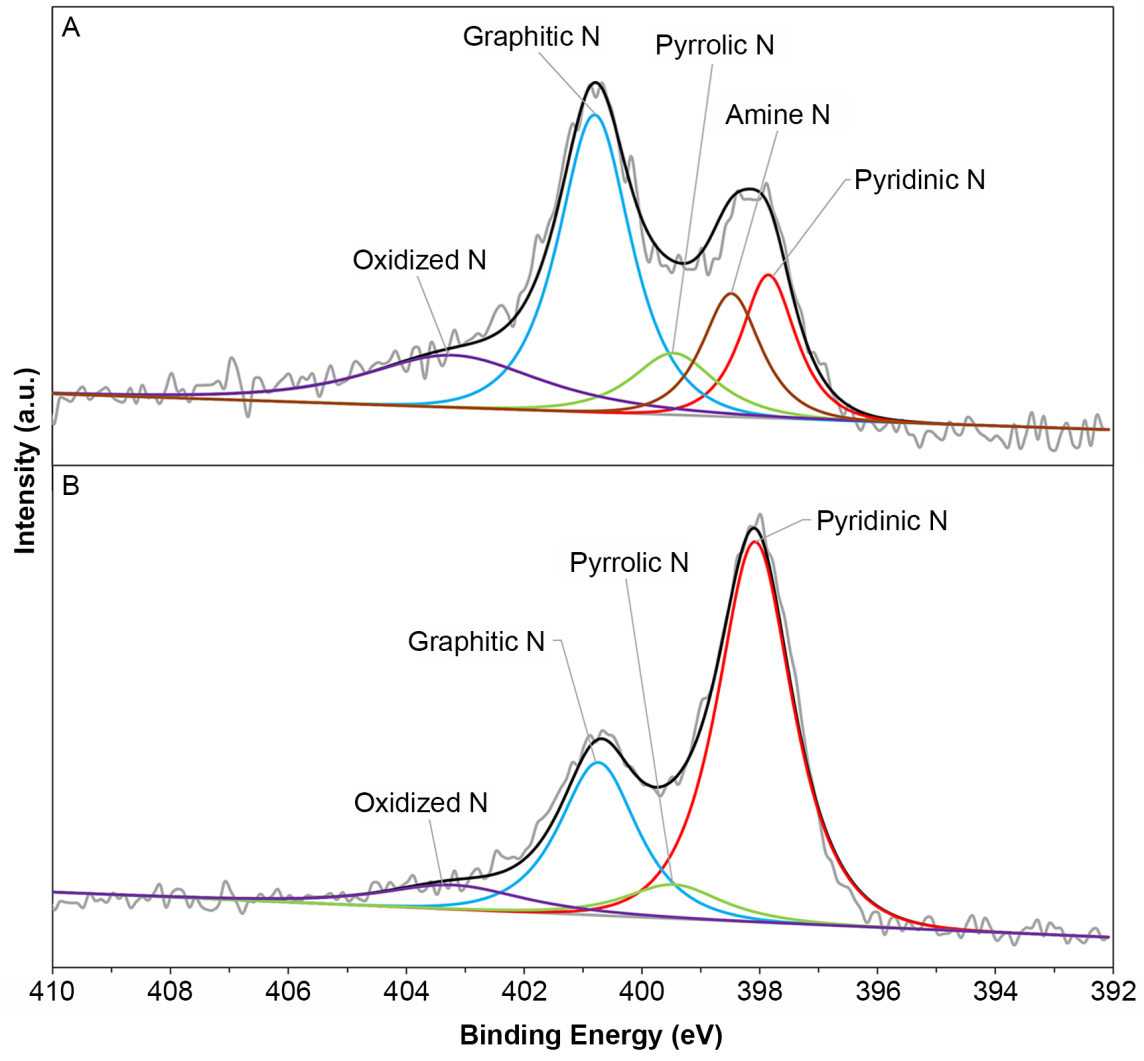
N 1s XPS spectra of (A) CNS-E-600 and (B) CNS-600.

The surface chemistry and bulk chemical composition were investigated using XPS, EDX, and Raman spectroscopy. The XPS surveys indicate that only carbon, oxygen, and nitrogen species were present in CNS-E-600 and CNS-600 (Figure S3A,D). This is consistent with the EDX mapping data that showed a uniform distribution of C, O, and N throughout the sample (Figure S4). The high-resolution C 1s spectra for both samples were remarkably similar and displayed a single, asymmetrical peak slightly above 284 eV, typical for carbon materials containing graphitic carbon (Figure S3B,E). The C 1s spectra were deconvoluted into C=C (284.28 eV), C–C (284.78 eV), sp^2^ C–N/O (285.48 eV), sp^3^ C–N/O (286.18 eV), and C=O (287.68 eV) species.^28−31^ A broad π–π* shakeup peak (290.98 eV) was also observed in both samples, which corresponds to a secondary emission of the conjugated aromatic C=C peak.^32^ The high-resolution O 1s spectra were deconvoluted into C=O (531.98 eV), sp^3^ C–O (532.58 eV), sp^2^ C–O (533.08 eV), and N=O (534.08 eV) species (Figure S3C,F).^30,31^ Interestingly, the sample prepared with EDTA showed a higher ratio of all oxygen species, except sp^3^ C–O (Table S2). A major source of sp^3^ C–O can be attributed to the reactive hydroxymethylphenol end groups formed during the initial polymerization (Figure 1). However, as polymerization proceeds, the end groups further cross-link with other aromatic species to form methylene bridges. Thus, the decreased ratio of sp^3^ C–O observed in the EDTA-assisted sample is indicative of extensive cross-linking, which is consistent with our observation in Figure S1, where the addition of EDTA led to faster reaction kinetics. The high-resolution N 1s spectrum for CNS-E-600 in Figure 5A could be deconvoluted into pyridinic-N (397.8 eV), amine-N (398.5 eV), pyrrolic-N (399.5 eV), graphitic-N (400.8 eV), and oxidized-N (403.2 eV) species.^12,22,34,35^ Similar N species were observed for the control sample except that no amine peak was present (Figure 5B).

**Scheme 1:**
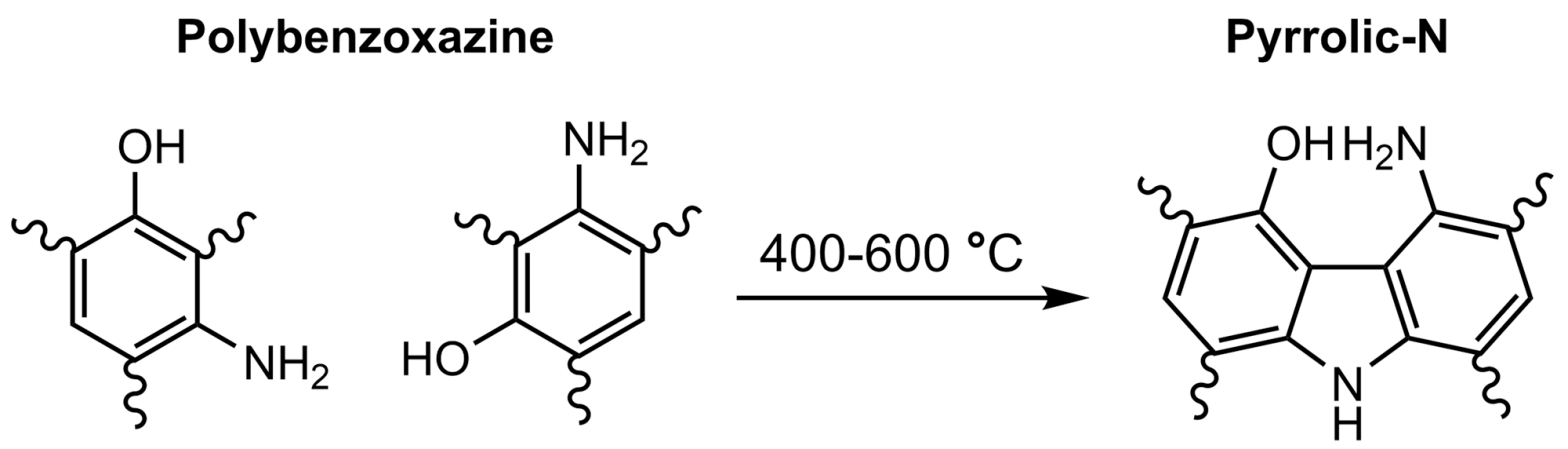
Formation of Pyrrolic-N from Polybenzoxazine Species

Amine groups are usually generated during the curing process, where ring-opening polymerization of benzoxazine generates arylamine and hydroxyl groups.^36,37^ However, these groups quickly react during carbonization at 400 °C, through an oxidative coupling reaction to produce pyrrolic-N groups (Scheme 1).^38,39^ At temperatures above 600 °C, pyrrolic-N is consecutively converted to pyridinic-N and then to graphitic-N and oxidized-N. Amine groups originating from benzoxazine were not observed at high carbonization temperatures, consistent with CNS-600. Thus, the amine peak observed in CNS-E-600 must have originated from EDTA. It is important to note that amine species are often confused with pyridinic-N, which can be observed up to 398.3 eV when located next to an edge or defect.^35^ Therefore, in order to better differentiate the two species, Raman spectroscopy was used to evaluate the concentration of defects.

**Figure 6:**
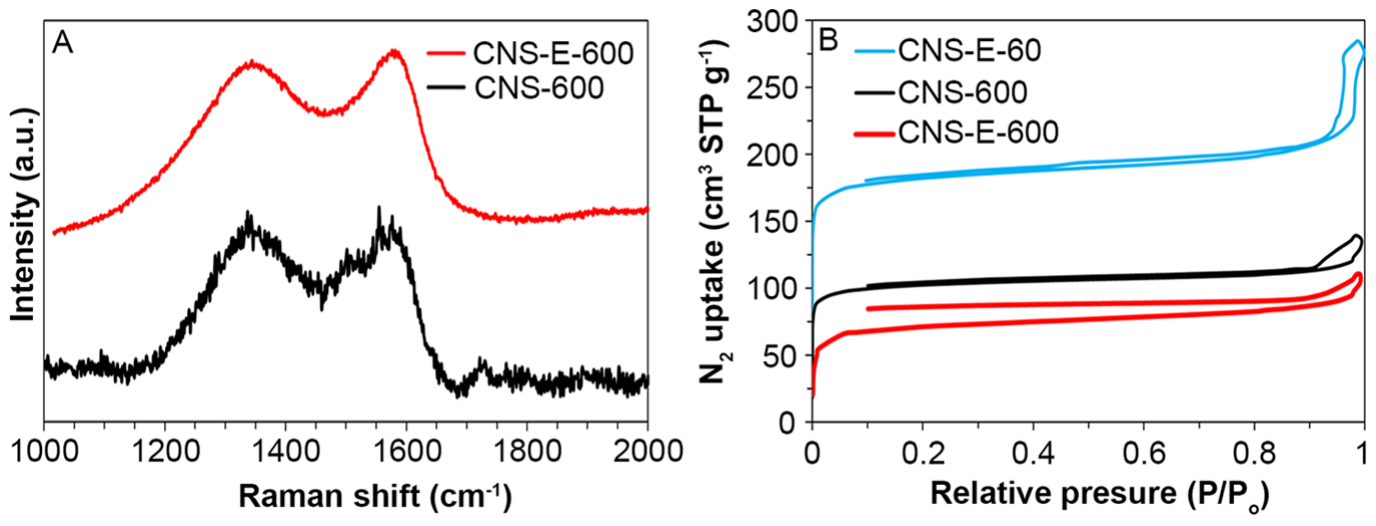
Combination of (A) Raman spectra and (B) nitrogen adsorption–desorption isotherms.

As shown in Figures 6A and S5, the Raman spectra of samples prepared with and without EDTA both displayed two peaks around 1340 and 1570 cm^–1^, which correspond to the D and G band, respectively. The ratio between these peaks (*I*_D_/*I*_G_) is often used to evaluate the degree of graphitization, as the D band is related to the amount of defects (i.e., amorphous carbon and discontinuities in the graphitic structure), while the G band corresponds to the ideal graphite lattice.^28^ The *I*_D_/*I*_G_ ratio of CNS-E-600 was 0.96, slightly lower than the value of 1.00 observed for CNS-600, indicating that the sample prepared with EDTA actually had fewer defects than the sample prepared without EDTA. Considering CNS-E-600 had a lower defect concentration than CNS-600, it was unlikely that the N 1s XPS peak located at 398.5 eV in Figure 5A originated from the pyridinic-N located near a defect. Rather, it is probable that this peak was actually an amine peak originating from the tertiary amine in EDTA.^40,41^

We used N_2_ sorption analysis to determine the specific surface area and porosity (Figure 6B and Table S3). All of the samples exhibited a type IV isotherm with hysteresis. CNS-E-600 had a specific surface area of 232 m^2^ g^–1^, which was lower than the value of 329 m^2^ g^–1^ observed for CNS-600. Despite having a lower surface area, the micropore volume of CNS-E-600 increased relative to CNS-600, suggesting that EDTA might be capable of increasing the surface roughness through micropore formation. Furthermore, CNS-E-60 showed the highest surface area at 579 m^2^ g^–1^, which could be attributed to the small particle size and increased roughness. Interestingly, CNS-E-60 displayed a notable hysteresis above 0.9 *P*/*P*_0_, despite not having any mesoporosity. This is often a consequence of closely packed structures, where N_2_ condenses in the interspatial cavities between nanospheres, resulting in dramatic N_2_ uptake.^42^ However, this was not observed for CNS-E-600 as the interspatial cavities were too large for N_2_ condensation.

Our TGA/DSC studies indicated that both samples had high thermal stability, approaching 400 °C before decomposition occurred (Figure S6). Elemental analysis confirmed that the nitrogen content increased from 4.97 to 6.24 wt % when EDTA was introduced into the synthesis (Table S4). Interestingly, the nitrogen in CNS-E-600 primarily existed as graphitic-N at a high level of 42.66%, whereas the control sample, CNS-600, only contained 25.24% (Table S2). Although there is some controversy, many reports have demonstrated graphitic-N to be the most promising species toward ORR and OER.^16,18,34^ Taken together, in addition to acting as an emulsion stabilizer, EDTA may also serve as a new source to functionalize carbon nanospheres for electrocatalysis.

**Figure 7:**
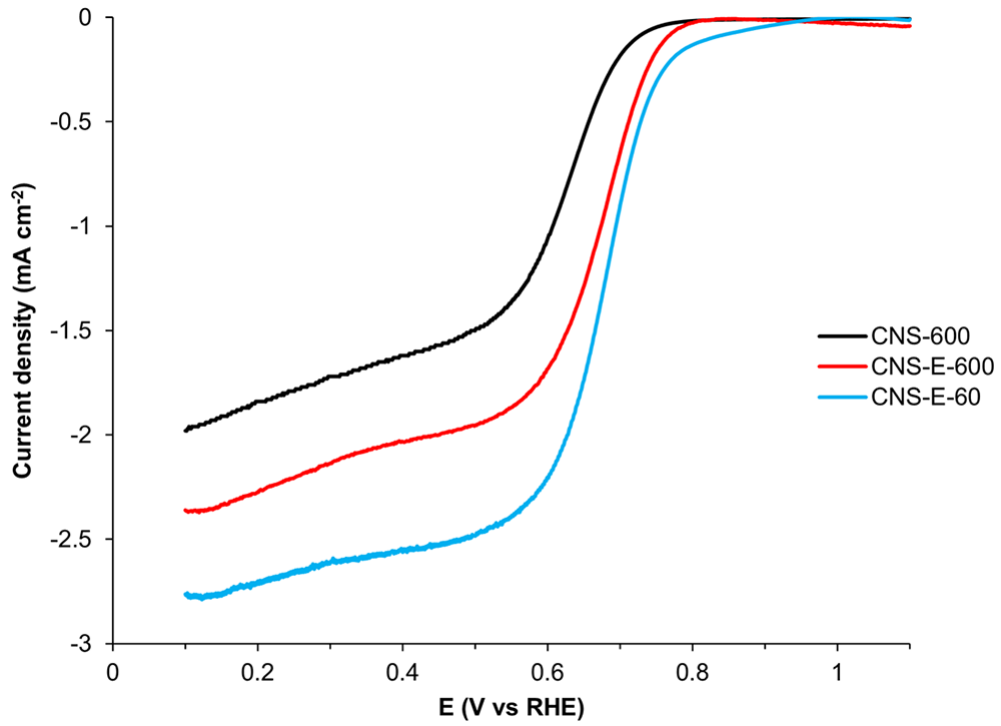
Combination of positive sweeping ORR polarization curves recorded in O_2_-saturated 0.1 M KOH. All ORR polarization curves have been corrected for double-layer capacitance.

The electrocatalytic performance of the prepared catalysts was evaluated for ORR in O_2_-saturated 0.1 M KOH. It is well-known that both surface functionalization and surface area affect heterogeneous catalysis. Thus, in order to investigate the effects of EDTA exclusively on surface functionalization, comparisons were made between CNS-E-600 and CNS-600, as they have similar particle sizes and surface areas. As evident by the ORR polarization curves in Figure 7, CNS-E-600 exhibited a more positive onset potential (*E*_onset_ = 0.82 V_RHE_) compared to the control sample (*E*_onset_ = 0.78 V_RHE_), proving that EDTA-assisted samples possessed superior ORR active sites. This trend agrees with the XPS data, which indicated CNS-E-600 contained a higher level of catalytically active graphitic-N sites. In general, diffusion limitations severely limit the maximum current density allowed for reactions involving gaseous reactants.^43^ ORR is no exception, and thus a diffusion-limited current density was observed at 2.37 mA cm^–2^ for CNS-E-600; however, CNS-600 was only capable of reaching 1.98 mA cm^–2^. The difference likely resulted from the uniform close-packing assembly observed for CNS-E-600, which contains periodic interspatial channels to enable facile mobility throughout the ordered structure. To further investigate the O_2_ diffusion dependence, LSV curves were acquired at various rotation rates between 400 and 2025 rpm (Figure S7). The corresponding Koutecky–Levich (K–L) plots derived from these LSV curves were both linear and parallel, implying that the ORR is indeed a diffusion-controlled first-order reaction (Figure S8). Furthermore, the average electron transfer number (*n*) calculated using the K–L equations (eqs S1 and S2) at various potentials was 2.00, confirming that ORR proceeds through a 2e^–^ pathway for the production of hydrogen peroxide.

Next, to demonstrate the full capacity of the sub-200 nm N-doped carbon nanospheres, the ORR activity of CNS-E-60 was also investigated. Astoundingly, the *E*_onset_ and diffusion-limited current density increased to 0.84 V_RHE_ and 2.76 mA cm^–2^, respectively, making CNS-E-60 a very promising metal-free ORR electrocatalyst (Figure 7). The K–L plots obtained for CNS-E-60 were also linear and parallel, suggesting that ORR was still a first-order reaction with respect to the dissolved O_2_ (Figures S9 and S10). However, the average electron transfer number (*n*) increased to 2.39, meaning that the 4e^–^ pathway was becoming more favorable. Because CNS-E-60 and CNS-E-600 have the same chemical composition, the deviation from the 2e^–^ pathway to 4e^–^ pathway suggests a dependence between the particle size and ORR mechanism. One possible explanation could be that CNS-E-60 had a higher specific surface area; therefore, the generated H_2_O_2_ might have a larger probability to interact with additional active sites before leaving the catalyst and thus being further reduced into water. This agrees with a recent report that has also observed a similar correlation between physical attributes and ORR selectivity.^44^

**Figure 8:**
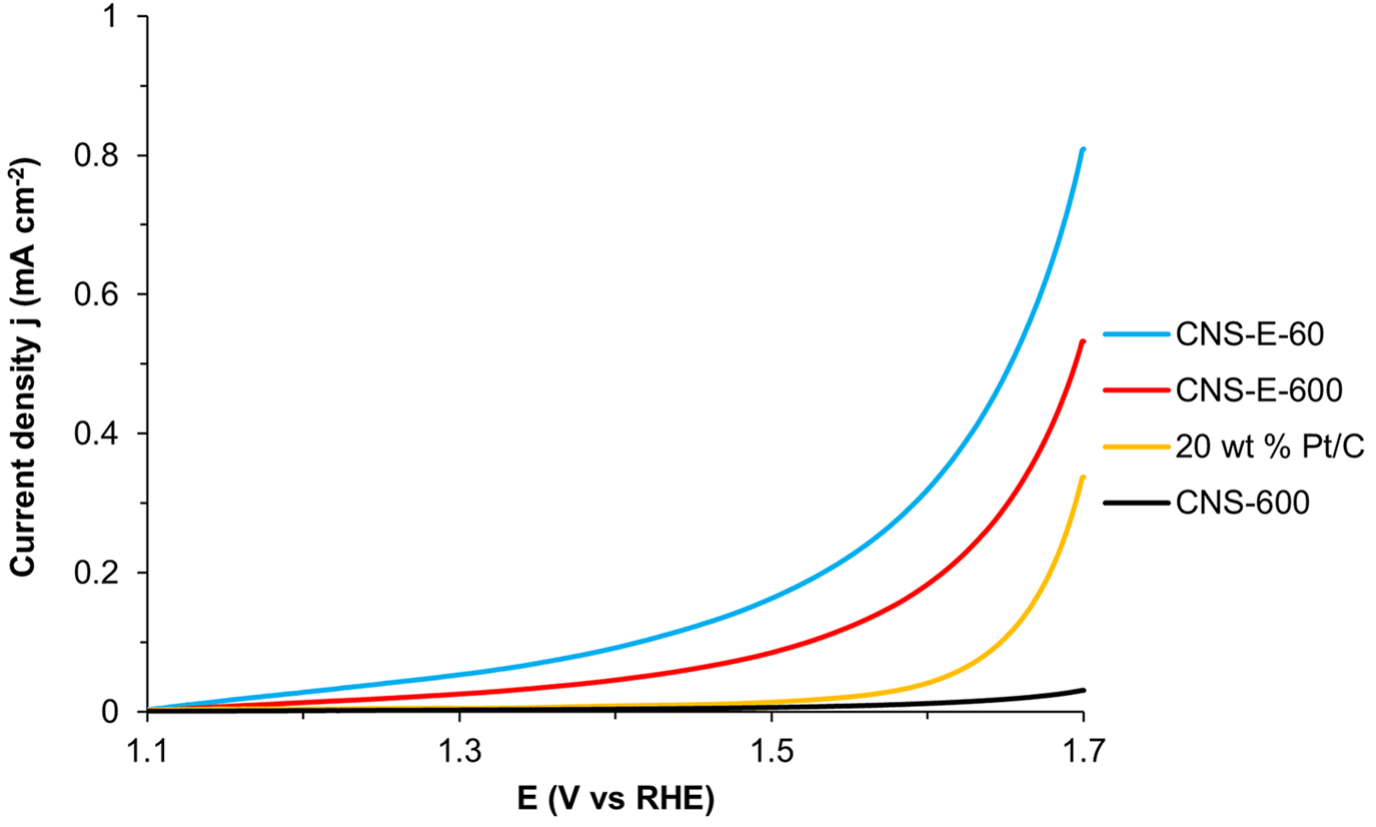
Comparison of OER polarization curves recorded in O_2_-saturated 0.1 M KOH.

To demonstrate the bifunctionality of the N-doped carbon nanospheres, we also evaluated the OER performance of the as-prepared catalysts in 0.1 M KOH. Similar to the trend observed for ORR, the samples prepared with EDTA were significantly more active than the counterparts prepared without EDTA (Figure 8). Again, by comparing the samples with similar surface areas, namely, CNS-E-600 and CNS-600, we can elucidate the impact that EDTA has on surface functionalization toward OER. Specifically, CNS-E-600 was capable of achieving an *E*_onset_ and current density (measured at 1.55 V_RHE_) of 1.38 V_RHE_ and 0.12 mA cm^–2^, respectively, which was significantly better than the 1.44 V_RHE_ and 0.0083 mA cm^–2^ observed for CNS-600, respectively. Additionally, CNS-E-600 eVen outperformed the commercial 20 wt % Pt/C. As expected, CNS-E-60 exhibited the highest activity among the samples tested, with an *E*_onset_ of 1.33 V_RHE_ and a current density of 0.22 mA cm^–2^. The enhanced catalytic performance of CNS-E-60 further confirms that the combination of high uniformity, N-functionalization, and sub-200 nm particle size is beneficial for metal-free OER electrocatalysis.

## Conclusions

In summary, we have successfully prepared N-doped carbon nanospheres as uniform samples using an EDTA-assisted method. In this protocol, EDTA acts as an emulsion stabilizer to prevent the droplets from coalescence into irregular structures via electrostatic repulsion. The diameters of the resultant N-doped carbon nanospheres could be readily tuned from 100 to 375 nm by controlling the EDTA and monomer concentrations. Without compromising monodispersity throughout this size range, it was possible to produce self-assembled opaline lattices with unique size-dependent optical properties. Other than the geometrical improvements, EDTA was also shown to increase the nitrogen content by ∼25%, which primary existed as graphitic-N, one of the most active functional groups for various electrochemical reactions. Benefiting from the reduced size, high uniformity, and surface functionalization, the as-prepared carbon nanospheres were demonstrated as efficient metal-free bifunctional electrocatalysts toward ORR and OER.

## Supporting Information Available

Summary of reaction conditions, photographs of reaction solutions, SEM, TEM, XPS, HAADF-STEM, EDX mapping, Raman, N_2_ sorption analysis, TGA, and electrochemical results (PDF)
