# Supplementary material for: EDTA-Assisted Synthesis of Nitrogen-Doped Carbon Nanospheres with Uniform Sizes for Photonic and Electrocatalytic Applications
Source: Chem Mater. 2023 Mar 28;35(7):3024–32. doi: 10.1021/acs.chemmater.3c00341 (PMC10100536; doi:10.1021/acs.chemmater.3c00341)
Supplement: Supplementary file 1 — cm3c00341_si_001.pdf [file cm3c00341_si_001.pdf]

Supporting Information for

EDTA-Assisted Synthesis of Nitrogen-Doped Carbon Nanospheres with Uniform Sizes for Photonic and Electrocatalytic Applications

Jacob Jeskey,[†] Yidan Chen,[§] Sujin Kim,[†] and Younan Xia[†,‡,*]

[†]School of Chemistry and Biochemistry, Georgia Institute of Technology, Atlanta, Georgia 30332, United States

[§]School of Materials Science and Engineering, Georgia Institute of Technology, Atlanta, Georgia 30332, United States

[‡]The Wallace H. Coulter Department of Biomedical Engineering, Georgia Institute of Technology and Emory University, Atlanta, Georgia 30332, United States

[*]Corresponding author: younan.xia@bme.gatech.edu

Table S1. Summary of reaction conditions, particle size, and corresponding specific surface area of the samples studied.

Sample	EDTA (mg)	3-aminophenol (mg)	Formaldehyde (mL)	Diameter (nm)		Surface area ($m^2 g^{-1}$)
				Polymer nanospheres	Carbon nanospheres	
**EDTA study**						
CNS-60	0	60	0.036	113 ± 32	85–122	-
CNS-E-60-1	10	60	0.036	128 ± 7	103 ± 8	-
CNS-E-60-3	30	60	0.036	157 ± 5	143 ± 7	-
CNS-E-60	50	60	0.036	228 ± 18	178 ± 8	579
**Monomer study**						
CNS-E-75	50	75	0.045	256 ± 9	204 ± 8	-
CNS-E-100	50	100	0.060	284 ± 5	235 ± 6	-
CNS-E-600	50	600	0.36	464 ± 13	375 ± 4	232
CNS-600	0	600	0.36	382 ± 49	254–345	329

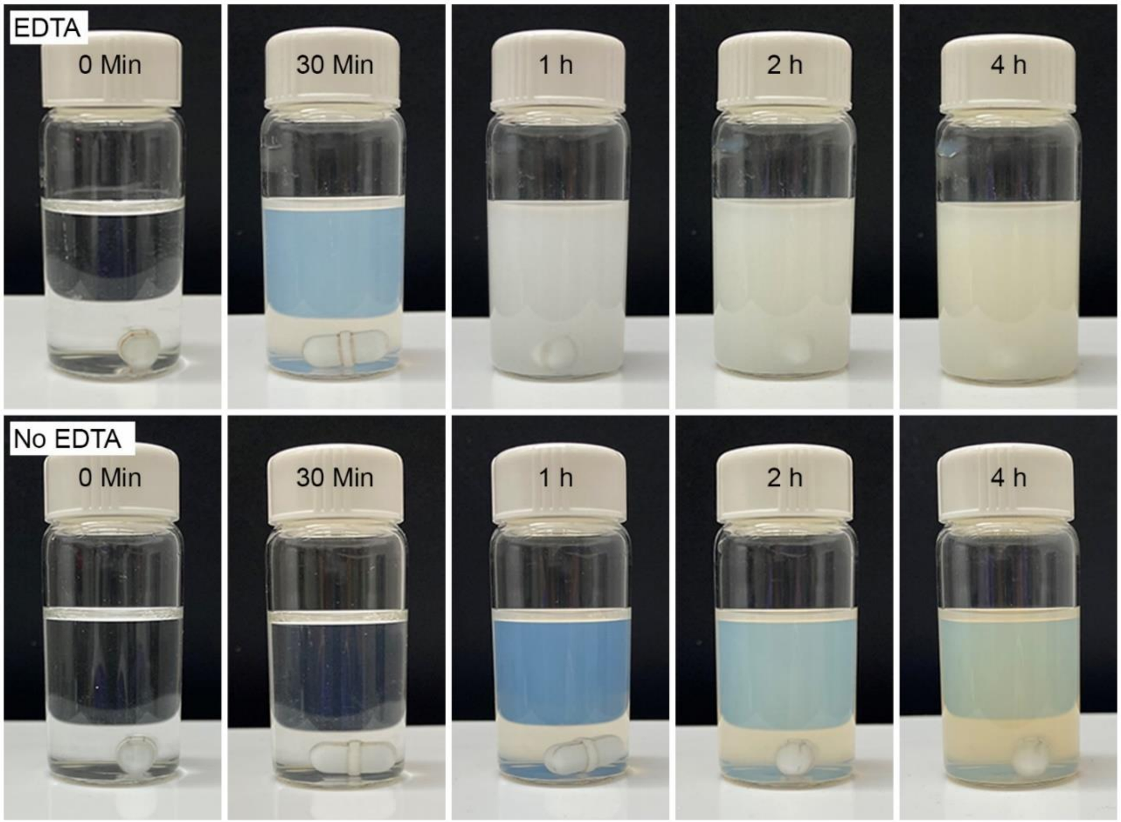

Figure S1. Photographs of the reaction solutions for (Top) CNS-E-60 and (Bottom) CNS-60, at different time points to show the color changes.

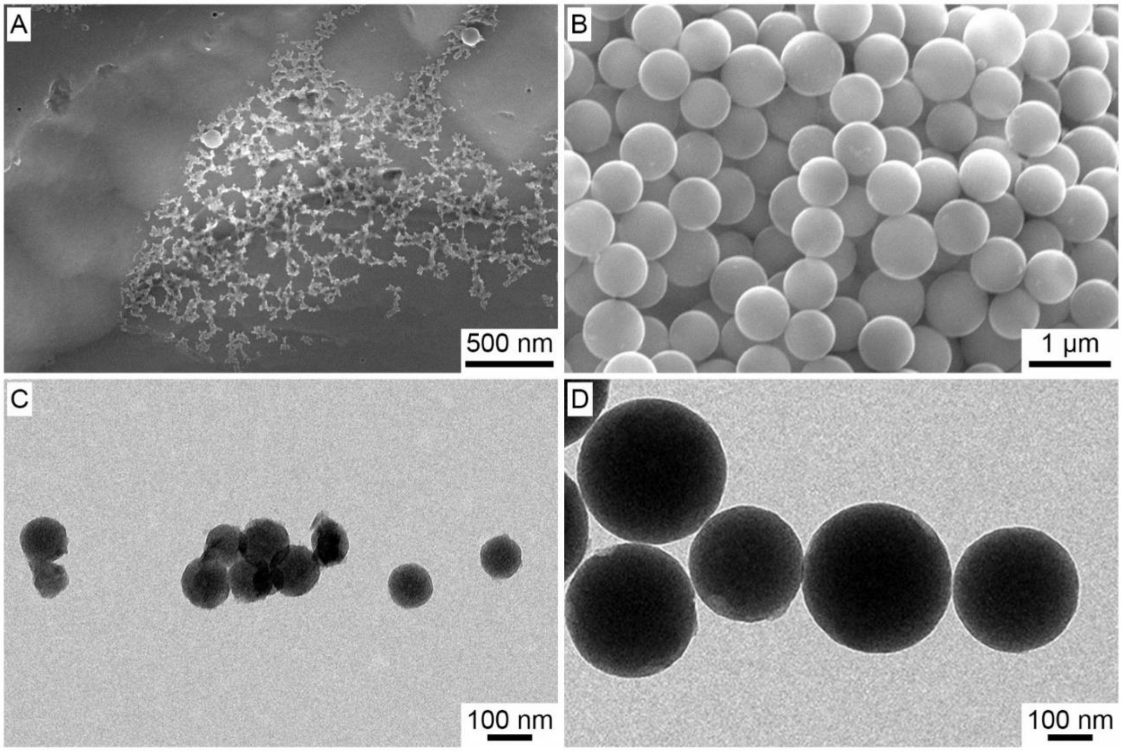

Figure S2. (Top) SEM and (Bottom) TEM images of control samples prepared in the absence of EDTA. The images in (A and C) correspond to CNS-60, the control sample prepared using the standard procedure without involving EDTA. The images in (B and D) correspond to CNS-600, where the amount of 3-aminophenol was increased to 600 mg.

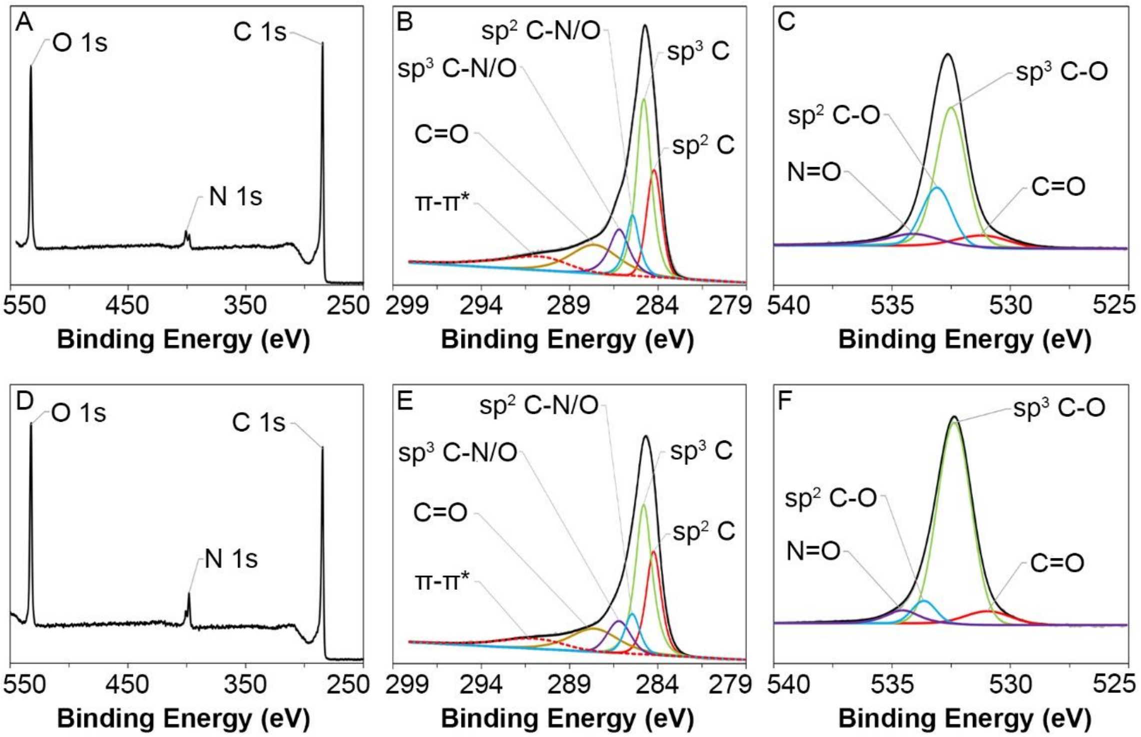

Figure S3. XPS spectra of (Top) CNS-E-600: (A) survey, (B) high-resolution C 1s, and (C) high-resolution O 1s; XPS spectra of (bottom) CNS-600: (D) survey, (E) high-resolution C 1s, and (F) high-resolution O 1s.

Table S2. Analysis of deconvoluted C1s, O 1s, and N 1s peaks.

Sample	C 1s relative area %					
	Sp^2 C	Sp^3 C	Sp^2 C–N/O	Sp^3 C–N/O	C=O	π–π*
CNS-E-600	18.97	36.96	9.62	10.97	6.63	16.84
CNS-600	19.60	33.55	9.63	10.23	6.41	20.58

	O 1s relative area %			
	C=O	Sp^3 C–O	Sp^2 C–O	N=O
CNS-E-600	8.67	57.15	23.05	11.13
CNS-600	7.07	79.74	7.03	6.15

	N 1s relative area %				
	Pyridinic	Graphitic	Pyrollic	oxidized	Amino
CNS-E-600	15.29	42.66	9.97	17.94	14.14
CNS-600	60.67	25.24	6.86	7.23	-

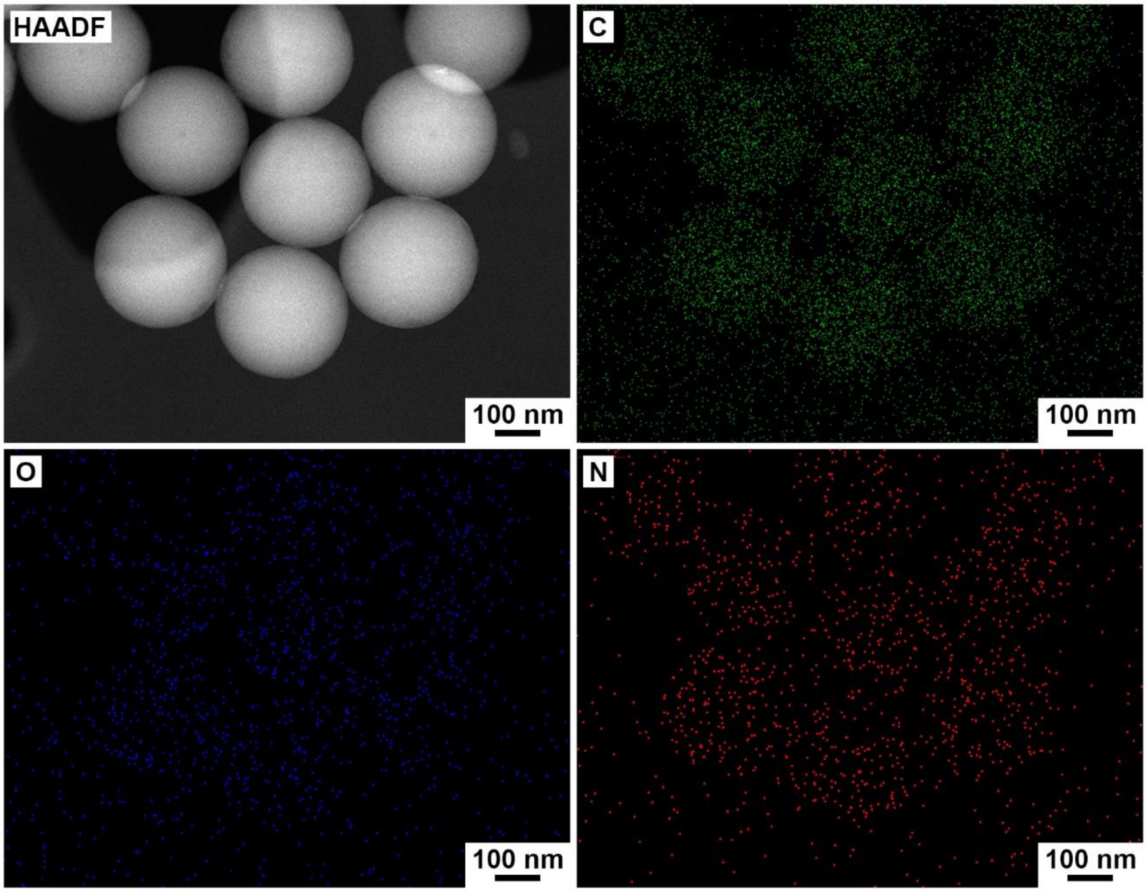

Figure S4. HAADF image and the corresponding EDX mapping of CNS-E-600.

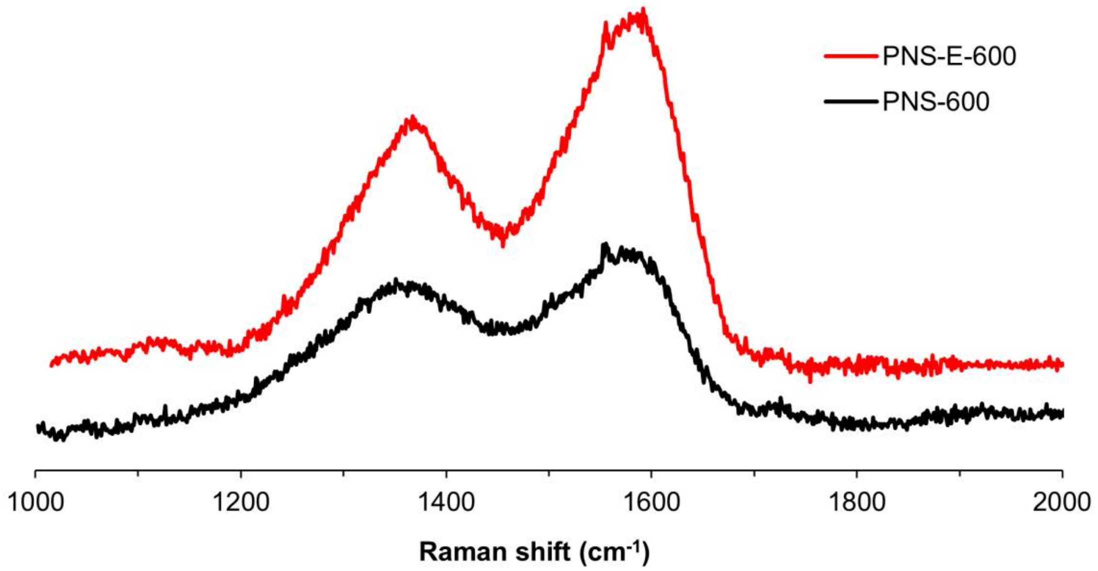

Figure S5. Raman spectra recorded from the polymer nanospheres before carbonization.

Table S3. Specific surface area and pore volume.

Sample	Specific surface area (m^2 g^{-1})	Micropore volume (cm^3 g^{-1})	Mesopore volume (cm^3 g^{-1})	Total volume (cm^3 g^{-1})
CNS-E-60	579	0.095	0.003	0.112
CNS-E-600	232	0.048	0.025	0.080
CNS-600	329	0.035	0.027	0.068

Table S4. Surface atom composition measured by XPS and bulk atom composition measured by elemental analysis for samples prepared in the presence/absence of EDTA.

Sample	XPS analysis (atomic %)			Elemental analysis – (wt %)			
	C	N	O	C	N	O	H
CNS-E-600	53.75	7.81	38.42	76.41	6.24	16.40	0.95
CNS-600	52.08	7.76	40.15	78.90	4.97	15.30	0.83

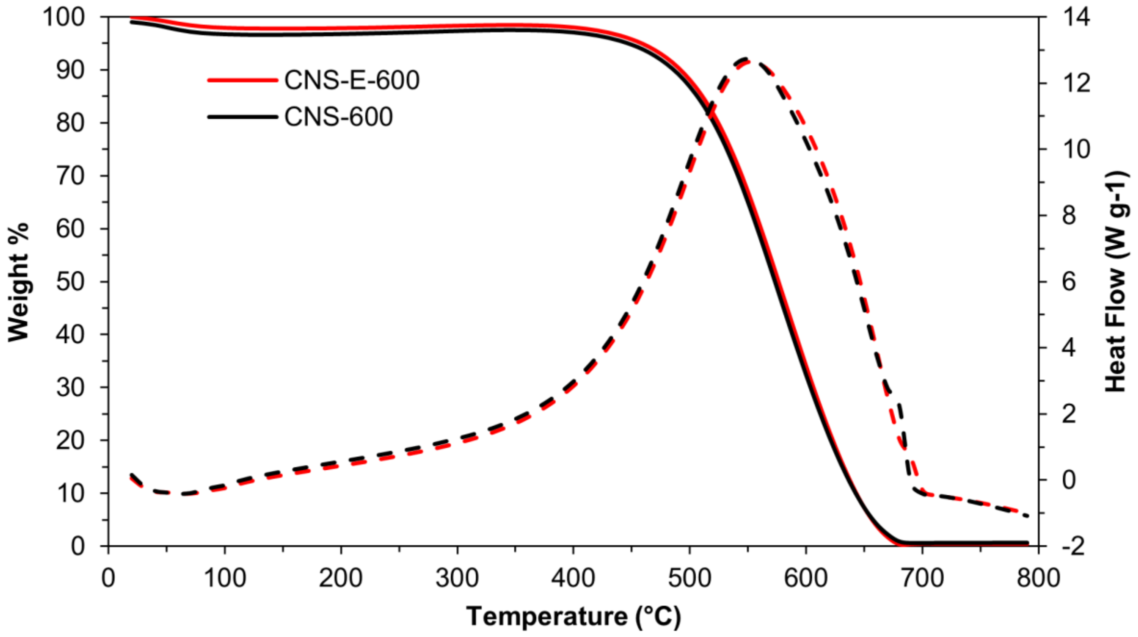

Figure S6. TGA and the corresponding DSC curves of CNS-E-600 and CNS-600 in air.

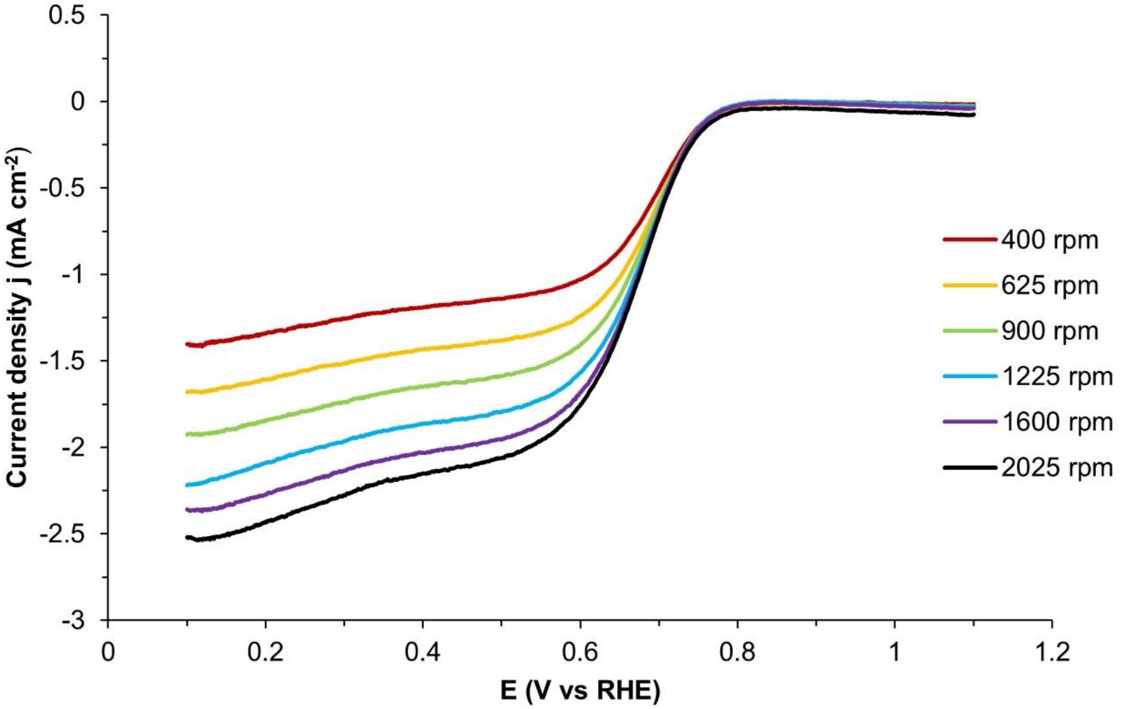

Figure S7. Positive sweeping LSV curves of CNS-E-600 at various rotating speeds recorded in O_2-saturated 0.1 M KOH.

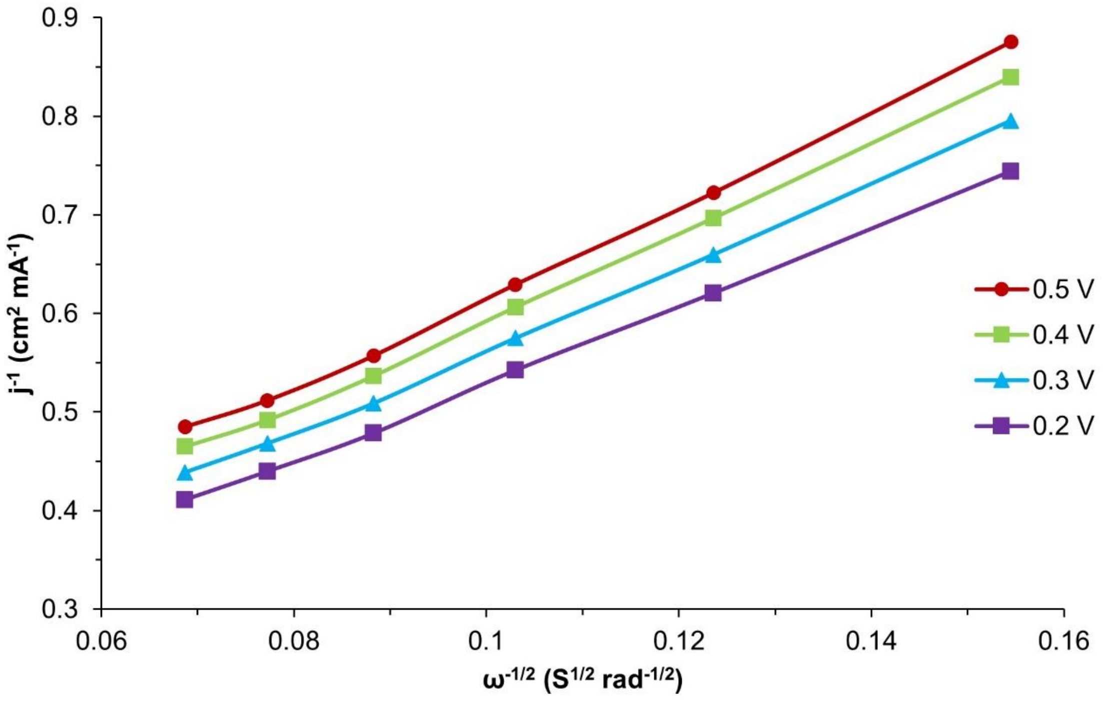

Figure S8. Koutecky–Levich (K–L) plots of CNS-E-600 at various potentials. The slopes indicate a $2e^{-}$ pathway. The K–L plots are both linear and parallel, reflecting that the ORR is indeed a diffusion-controlled first-order reaction.

$$\frac{1}{j} = \frac{1}{\beta\omega^{\frac{1}{2}}} + \frac{1}{j_K} \quad \text{(S1)}$$

$$\beta = 0.62nFD_0^{\frac{2}{3}}\mathrm{v}^{\frac{-1}{6}}C_0 \quad \text{(S2)}$$

The K–L equations above were used to calculate the transferred electron numbers (n), where j and j_K are the measured current density and the kinetic current density, respectively; ω is the electrode rotation speed; F is the Faraday constant (96485 C mol^{-1}); D_0 is the diffusion coefficient of O_2 (1.9 x 10^{-5} cm^2 s^{-1} for 0.1 M KOH; ν is the kinetic viscosity (0.01 cm^2 s^{-1}); and C_0 is the concentration of O_2 (1.2 x 10^{-6} mol cm^{-3}).

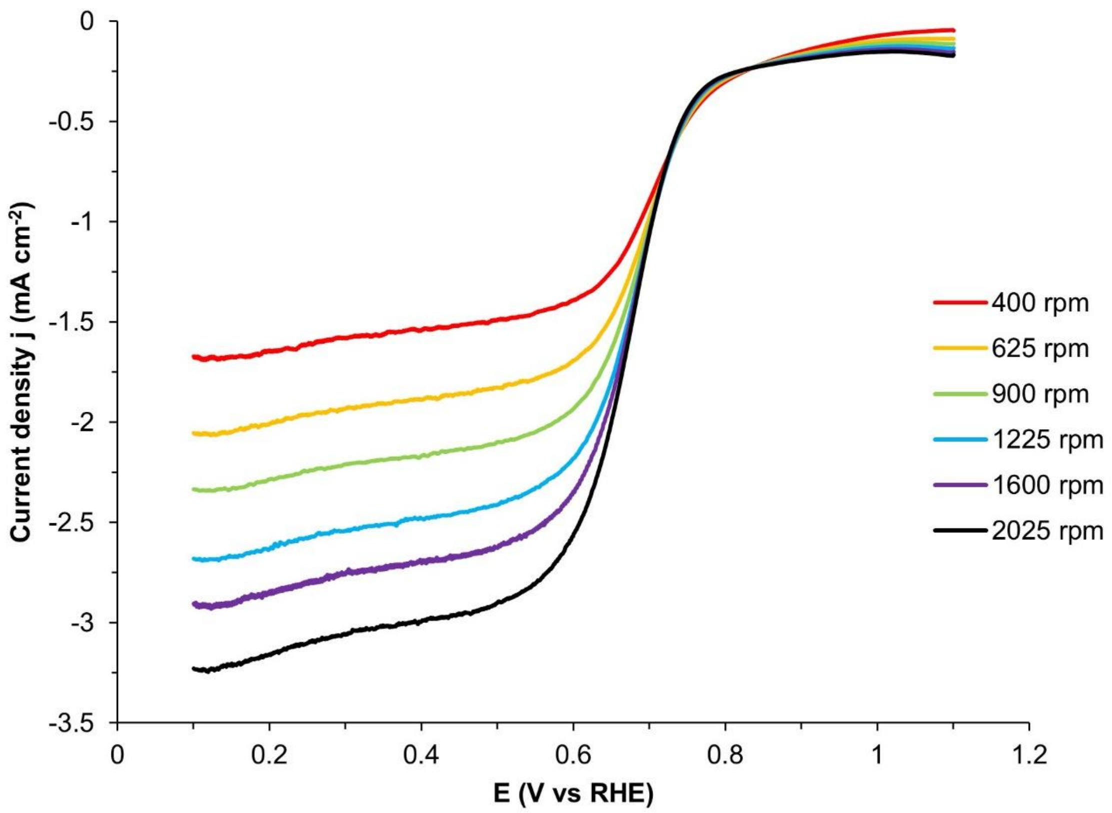

Figure S9. Positive sweeping LSV curves of CNS-E-60 at various rotating speeds recorded in O_2-saturated 0.1 M KOH.

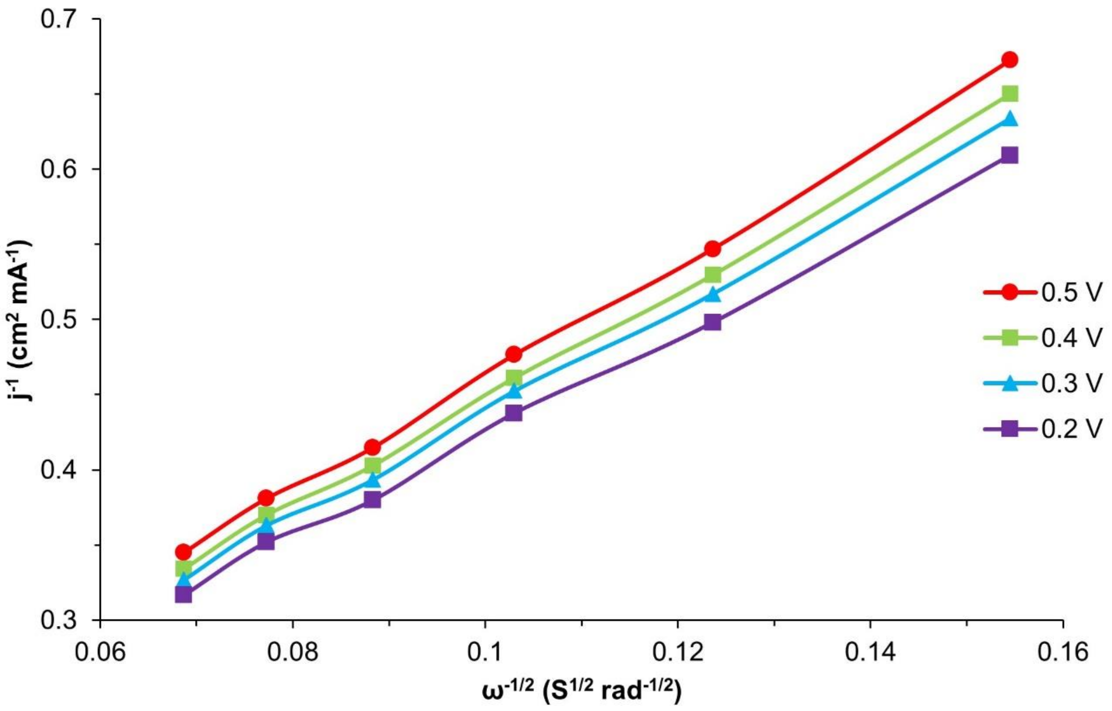

Figure S10. Koutecky–Levich (K–L) plots of CNS-E-60 at various potentials. Based on the slopes, the average electron transfer (n) was determined to be 2.39.
